# SWAV: a web-based visualization browser for sliding window analysis

**DOI:** 10.1038/s41598-019-57038-x

**Published:** 2020-01-10

**Authors:** Zhenglin Zhu, Yawang Wang, Xichuan Zhou, Liuqing Yang, Geng Meng, Ze Zhang

**Affiliations:** 1 School of Life Sciences, Chongqing University, No. 55 Daxuecheng South Rd., Shapingba, Chongqing, 401331 China; 2 College of Veterinary Medicine, China Agricultural University, Beijing, 100094 China; 3 The School of Microelectronics and Communication Engineering, Chongqing University, Chongqing, 400044 China; 4 Department of Medical Ultrasonics, Chongqing Occupational Disease Prevention Hospital, Chongqing, 400060 China; 5 Khoury College of Computer Sciences, Northeastern University, Seattle, 98109 WA USA

**Keywords:** Data publication and archiving, Software

## Abstract

Sliding window analysis has been extensively applied in evolutionary biology. With the development of the high-throughput DNA sequencing of organisms at the population level, an application that is dedicated to visualizing population genetic test statistics at the genomic level is needed. We have developed the sliding window analysis viewer (SWAV), which is a web-based program that can be used to integrate, view and browse test statistics and perform genome annotation. In addition to browsing, SAV can mark, generate and customize statistical images and search by sequence alignment, position or gene name. These features facilitate the effectiveness of sliding window analysis. As an example application, yeast and silkworm resequencing data are analyzed with SWAV. The SWAV package, user manual and usage demo are available at http://swav.popgenetics.net.

## Introduction

Sliding window analysis is an application in which test statistics are plotted with a sliding window at a certain length along a sequence or chromosome^1^; this type of analysis is ubiquitously employed to study the properties of chromosome sequences. To trace selective constraints, the manual inspection of the plotted statistics is helpful. A peak or valley in the plot may infer selection evidence in evolutionary biology. In traditional sliding window analysis, test statistics are plotted at one specific locus in R or Excel at each instance in time. If a large number of target loci exist, the workload is extensive. Additionally, the traditional test plotting methods do not include gene annotation. To assess the peripheral effects of one gene/sequence, the peripheral genes must be marked in the plot. Moreover, a genomic-scale browser is needed to view the test statistics in whole-genome or multitarget sliding window analysis.

In this paper, we describe a sliding window analysis viewer (SWAV) that enables users to rapidly export their statistical data, such as theta^2^, Fst^3^, Tajima’s D^4^ and the composite likelihood ratio (CLR)^5,6^, and simultaneously view gene annotation information and test statistics at various scales. Unlike the developed stand-alone programs^7–10^ for genome visualization, SWAV is designed for sliding window analysis. SWAV can integrate multiple test statistics in a track and plot the corresponding curves, such as in R. Furthermore, SWAV has special functions, such as marking focus regions, providing customization, exporting statistical images, and searching by position or the gene name. Because SWAV is highly specialized for sliding window analysis, it excludes unrelated functions and has a simple setup. Notably, SWAV not only accepts formatted data from the UCSC Genome Browser^10^ and Ensembl^11^ but also customizes newly generated data. Users can process and analyze special-format data in SWAV after changing a few data processing scripts offered by SWAV. SWAV utilizes recent developments in JavaScript and PHP and is reliable and user friendly for biological researchers. For developers, SWAV is an open source program that can be easily customized.

### The design and functionalities of SWAV

**Figure 1:**
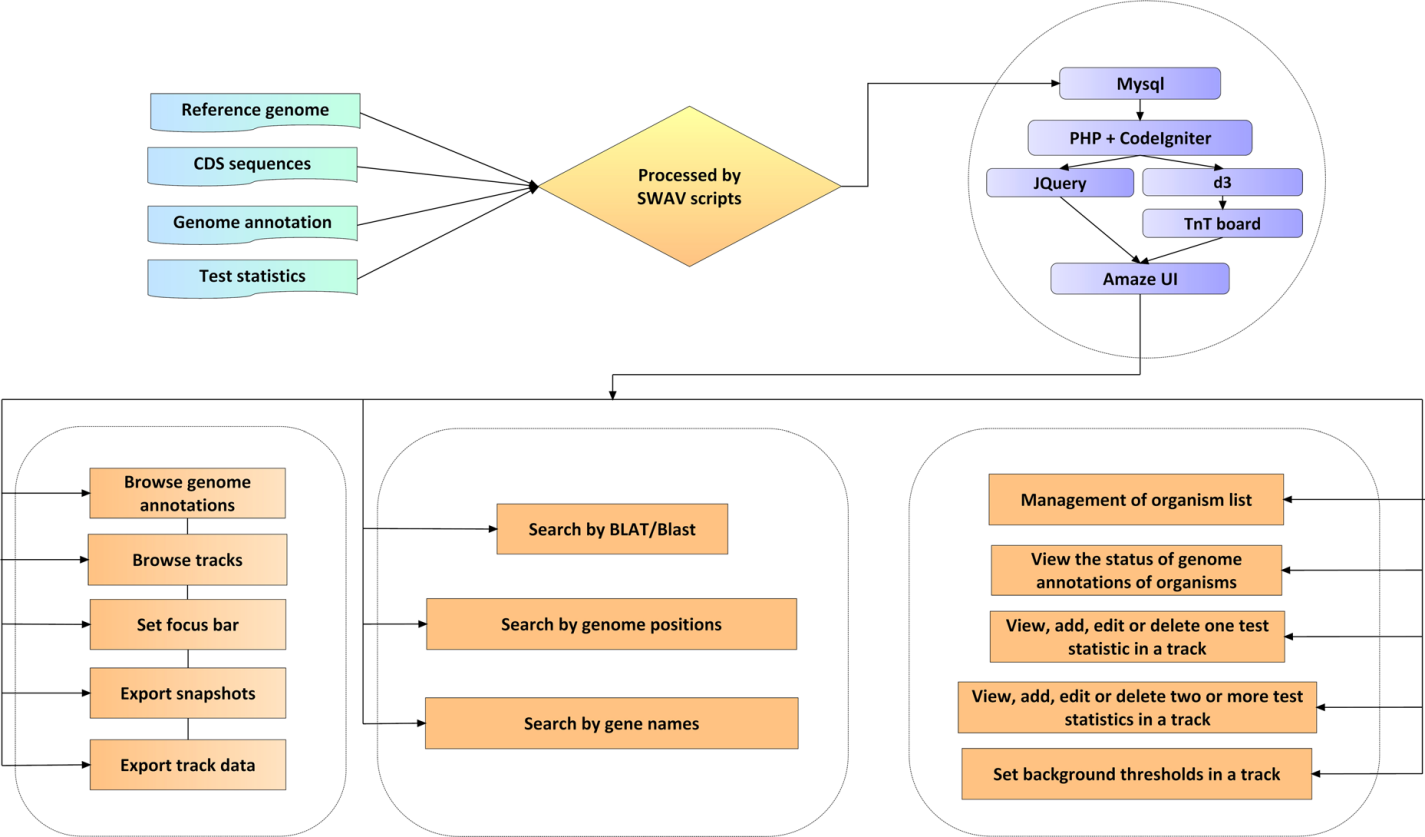
The workflow and main functions of SWAV.

The installation of SWAV is fast and easy and only requires the configuration of Apache and MySQL to provide an interactive visual panel (Fig. 1). After the SWAV codes are uploaded, users can add organisms in the setting panel and upload genome annotation data; then, a track of test statistics can be freely added or edited. SWAV offers scripts to process and upload genome annotation files in GFF or GTF format and test statistic files from ANGSD^12^ or other population analysis software packages. To facilitate observation and analysis, SWAV enables users to add more than one subtrack in a track viewer, and different subtracks can be plotted in different colors. The export and display of statistic data are simple (only two steps are needed) and fast. Initial users can spent less than 1 minute in average to finish the task. If users are familiar with the process, time consumption is reduced to nearly 30 seconds (Supplementary Table S1). SWAV also provides scripts to calculate background thresholds, which can be then added in the setting panel. There are only five steps required to set up SWAV. The detailed user manual is available at http://swav.popgenetics.net.

**Figure 2:**
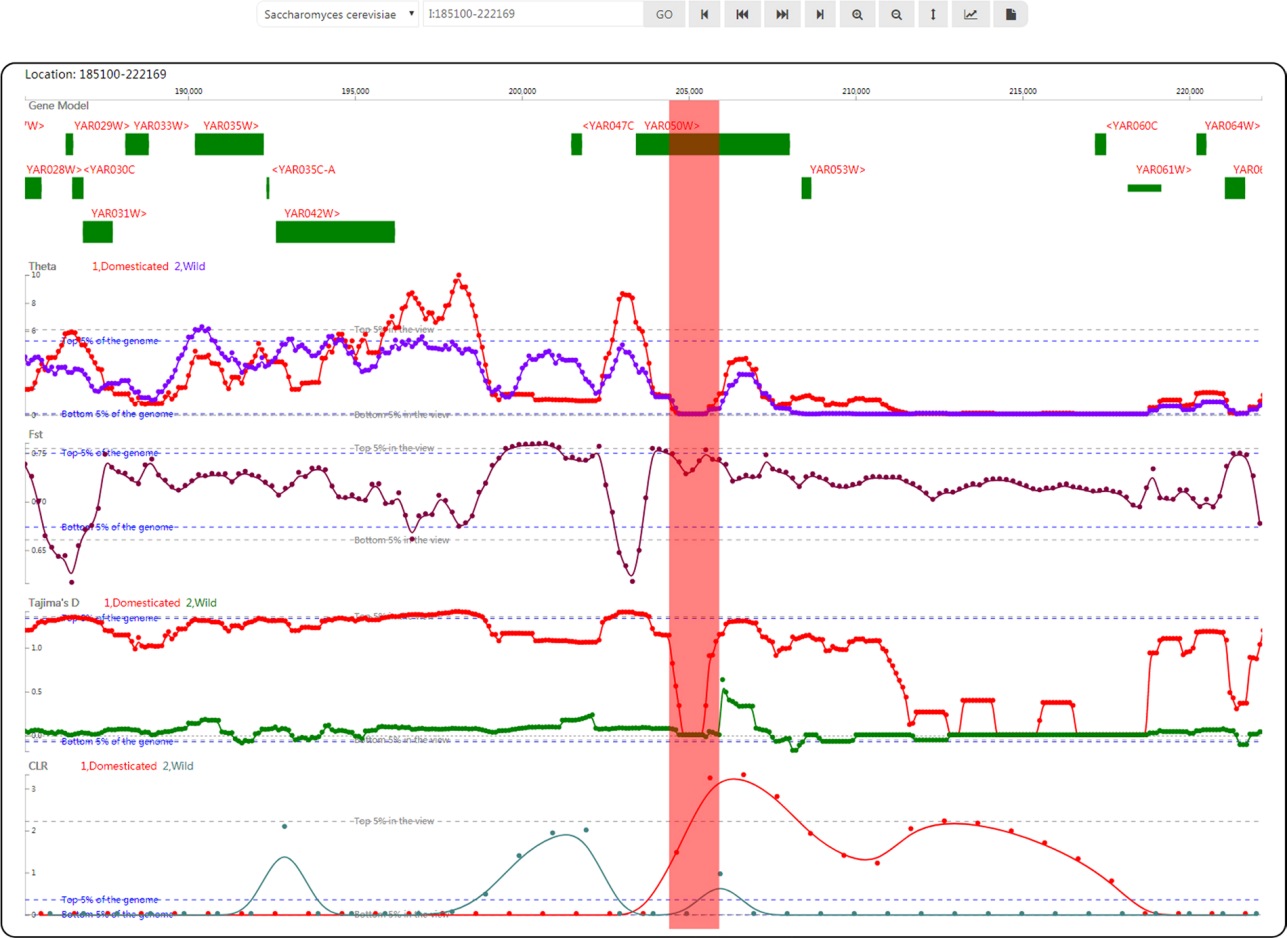
Snapshot of the viewer with genome annotation information and the tracks of test statistics. From the viewer, we observed a theta valley, an Fst plateau, a Tajima’s D valley and a CLR peak for Saccharomyces cerevisiae at YAR05W, as marked by the focus bar in red. The Fst plateau and CLR peak are both above the top 5% thresholds of the genome.

In the viewer, genome annotation information and the tracks of test statistics are interactively and successively listed in the center pane (Fig. 2). In sliding window analysis, background thresholds are usually employed to determine whether a region is selected. To this end, SWAV possesses 2 default thresholds (top and bottom 5% of the data in the view). The top and bottom 5% thresholds of the genome can also be calculated and displayed in the viewer. By choosing a specific position in the genome of an organism, users can easily view test values in the selected region and find regions of selective signatures.

SWAV also includes typical genome browser functions, such as panning and zooming in and zoom out. For sliding window analysis, SWAV has a focus bar function that enables users to mark a region in the viewer for detailed analysis. Users can customize and export specific plots of test statistics and download the statistical data in the viewer. SWAV offers BLAT^13^ searches for genomes or BLASTn^14^ searches for coding sequences. To facilitate multitarget analysis, SWAV retrieves regions using a list of genome positions or gene names.

We investigated an example application (swav.popgenetics.net/example) of SWAV by analyzing published yeast resequencing data (NCBI BioProject: PRJEB1973)^15^, including 3 domesticated samples (Saccharomyces cerevisiae) and 13 wild samples (Saccharomyces paradoxus). We mapped the reads of each sample onto the yeast reference genome (www.yeastgenome.org) with Bowtie2^16^ and calculated theta, Tajima’s D, and Fst in a window size of 1000 and with a step size of 100 using ANGSD. We also called the CLR of each chromosome using SweepFinder2^17^ based on the results from ANGSD. The threshold lines at 5% were plotted for all tracks. Taking YAR05W as an example, the positive selection signatures of this gene are clearly displayed in the SWAV genome viewer (Fig. 2). This gene encodes proteins with functions in mating and survival^18^. To test SWAV for higher eukaryotes, we applied SWAV to the population genetics analysis results of domestic silkworms and wild silkworms (NCBI BioProject: PRJDB4743). We utilized the updated genome annotation for the silkworm from the silkbase (http://silkbase.ab.a.u-tokyo.ac.jp/) in SWAV.

## Discussion

SWAV is designed to visualize and rapidly release genome test statistics on the web. This application accelerates the manual inspection process in sliding window analysis and simplifies the generation of statistical images. Compared to the custom track tool in the UCSC Genome Browser^10^, SWAV displays custom tracks with curves, but the UCSC Genome Browser displays custom tracks in blocks in different colors. Notably, curves are ideal for sliding window analysis. Furthermore, SWAV can display two or more types of test statistics in one track, which facilitates comparison and analysis. SWAV executes most processes with JavaScript and does not require CGI. Thus, must work is shifted from the server to the client, which can improve the overall performance. The abandonment of CGI makes the installation of SWAV simple and easy, considering that the construction of a CGI environment is difficult for most users. SWAV also provides several analysis tools specifically for sliding window analysis, such as the setting of customized thresholds for tracks, the setting of a focus bar in the viewer, the exporting of track plots, and the exporting of track data in the viewer. To facilitate multitarget analysis, SWAV also offers region retrieval using a list of genome positions or gene names.

The application of SWAV for yeast and silkworm analysis is only the start of research in this area. SWAV will be used for population genetic analyses of more organisms in the future.

### Implementation details

In the development of SWAV, we employed the updated version of the PHP framework CodeIgniter 3.1.9 (www.codeigniter.com), which is highly secure and maintainable. For front-end web coding, we utilized JQuery (jquery.com), d3 (d3js.org) and TnT broad (tntvis.github.io/tnt.board) as JavaScript libraries and the HTML5 framework AmazeUI (amazeui.org) as the background style to establish the graphic interface (Fig. 1).

## Supplementary information

Table S1.
